# Adult male mice exposure to nonylphenol alters courtship vocalizations and mating

**DOI:** 10.1038/s41598-018-21245-9

**Published:** 2018-02-14

**Authors:** Daphné Capela, Carlos Dombret, Kevin Poissenot, Manon Poignant, Aude Malbert-Colas, Isabelle Franceschini, Matthieu Keller, Sakina Mhaouty-Kodja

**Affiliations:** 1 Sorbonne Universités, UPMC Univ Paris 06, INSERM, CNRS, Neuroscience Paris Seine–Institut de Biologie Paris Seine, Paris, 75005 France; 2 Institut National de la Recherche Agronomique, UMR 85, Nouzilly, 37380 France; 3 Centre National de la Recherche Scientifique, UMR 7247, Nouzilly, 37380 France; 4 Université François Rabelais, Tours, 37000 France; 5 Institut Français du Cheval et de l’Equitation, Nouzilly, 37380 France

## Abstract

The neural circuitry processing male sexual behavior is tightly regulated by testosterone and its neural metabolite estradiol. The present study evaluated the effects of adult exposure to low doses of nonylphenol (NP), a widespread environmental contaminant, on the neuroendocrine regulation of testosterone and expression of sexual behavior. Oral exposure of C57BL/6J males to NP (0.5, 5 or 50 μg/kg/day) for 4 weeks did not affect circulating levels of testosterone or the kisspeptin system, a key regulator of the gonadotropic axis. In contrast, mice exposed to NP at 5 μg/kg/day emitted an increased number and duration of ultrasonic vocalizations, took longer to reach ejaculation and showed increased number of mounts, intromissions and thrusts. This was associated with normal olfactory preference and locomotor activity, and increased anxiety level. Analysis of the neural circuitry that underlies sexual behavior showed changes in the number of cells expressing androgen and estrogen receptors in males exposed to NP at 5 μg/kg/day. The neural circuitry underlying sexual behavior is thus highly sensitive to adult exposure to NP. Furthermore, almost all the observed effects were induced at 5 μg/kg/day of NP, indicating that this endocrine disrupter triggers a non-monotonic response in the adult male mouse brain.

## Introduction

Nonylphenol (NP) is an organic compound used in the manufacture of NP ethoxylate surfactants, but derives also from alkylphenol degradation that occurs during sewing water treatment or in the environment. Alkylphenols are nonionic surfactants that have been used since 1950 in a wide variety of industrial, agricultural and domestic applications such as soap, cosmetics, paints, herbicides and pesticides, or plastic fabrication. This results in major environmental contamination by NP of ecosystems including sewing water and rivers. NP was classified by the EU in 2000 as a priority substance “presenting a significant risk to or via the aquatic environment” in the Water Framework Directive 2000/60/EC, which was updated in 2008 and 2013^1^. Due to its widely-reported estrogenic activity, NP may affect male reproductive capacities as was highlighted in a recent systematic review^2^.

Male reproduction is controlled by finely regulated cross-talk between neural and peripheral structures. Synthesis and liberation of testosterone by the testes are under the control of hypothalamic gonadotropin-releasing hormone (GnRH) and pituitary luteinizing hormone (LH). In turn, testosterone acts on the male brain through androgen (AR) and estrogen (ER) receptor-dependent signaling pathways to regulate GnRH release, thereby controlling gonadotropin secretion and circulating testosterone levels. One key neuronal system that mediates the negative feedback exerted by testosterone and its neural metabolite estradiol on GnRH release is kisspeptin neurons of the arcuate nucleus^3^. The sexually dimorphic anteroventral periventricular area (AVPV) and periventricular (PeN) of the preoptic area contain much fewer kisspeptin neurons in males^4,5^. AR and ER signaling in kisspeptin neurons regulate transcription of the *Kiss1* gene, which encodes the potent GnRH secretagogue kisspeptin. The kisspeptin system has recently emerged as a key central target of endocrine disruptors that affect sex steroid signaling and reproductive function [see^6,7^ for a review].

At the behavioral level, testosterone also organizes perinatally and activates during adulthood the neural circuitry involved in the expression of sexual behavior. This complex behavior comprises pre-copulatory and copulatory phases. During the pre-copulatory (motivational) phase, the courting male displays a chemo-investigating behavior toward receptive females and produces ultrasonic vocalizations, which play a role in attracting the female partner^8^. During the copulatory (consummatory) phase, the male exhibits mounting, thrusting, and intromitting behavior before reaching ejaculation. Interference with testosterone-induced regulation of these neuroendocrine functions and behavior could disrupt male reproduction.

Studies in rats showed that gestational and postnatal exposure to NP lowered testosterone levels at NP doses ranging from 25 to 450 mg/kg/d^9–14^. Of these studies, two reported a parallel increase in LH and FSH (follicle stimulating hormone) levels^10,13^, suggesting a long-term effect on the hypothalamus-pituitary-gonad (HPG) axis. Interestingly, exposure of adult rats to NP doses between 100 μg/kg/d and 300 mg/kg/d was also found to reduce testosterone production^15–18^ and increase LH levels^15^, pointing out a vulnerability of this axis to high NP doses.

At the behavioral level, 25 studies (22 in rat and 3 in mice) addressed the effects of exposure to NP on the brain and on behavior. However, only one analysis evaluated the effects of exposure to NP on reproductive behavior. Nagao *et al*.^19^, reported that postnatal exposure to a high NP dose (500 mg/kg) had no effect on sexual behavior in rats. Two mouse studies evaluating the percentage of mating males, without a detailed analysis of behavior showed no effect of gestational or postnatal exposure to NP at doses ranging from 1.2 to 123 mg/kg/d^20,21^. In two neuroanatomical studies, the sexually dimorphic nucleus located in preoptic cells that plays a key role in sexual behavior was either increased or unaffected following gestational and postnatal exposure to NP at doses ranging from 25 to 3000 ppm^22,23^. In this context, no studies evaluated the potential effects of adult exposure to low doses of NP on male neuroendocrine and behavioral responses. Such an evaluation is particularly pertinent in light of our recent results that demonstrate a high sensitivity of adult neural circuits processing sexual behavior toward endocrine disruptors with estrogenic and anti-androgenic activities^24,25^.

The present study was undertaken to characterize the effects of chronic adult male mouse exposure to low doses of NP on the HPG axis and sexual behavior. For this purpose, male mice were orally exposed to the vehicle alone or to NP (50, 5 and 0.5 μg/kg/d) for four weeks. Testosterone levels and weight of androgen-sensitive tissues of the genital tract were compared between the four groups. Kisspeptin-immunoreactive neurons and co-expressed AR or ER were quantified in the AVPV and PeN subdivisions of the rostral preoptic periventricular nucleus located in the preoptic area, as well as in the arcuate nucleus. Courtship behavior of males was analyzed by testing olfactory preference and emission of courtship vocalization in the presence of receptive females. Latency and frequency of copulatory behavior were also quantified. Furthermore, we measured locomotor activity and anxiety-state level, which, if altered, could interfere with the expression of sexual behavior. Finally, the number of AR- and ER-immunoreactive neurons in the neural circuitry that underlies sexual behavior was determined.

## Results

### Analysis of the HPG axis

**Figure 1:**
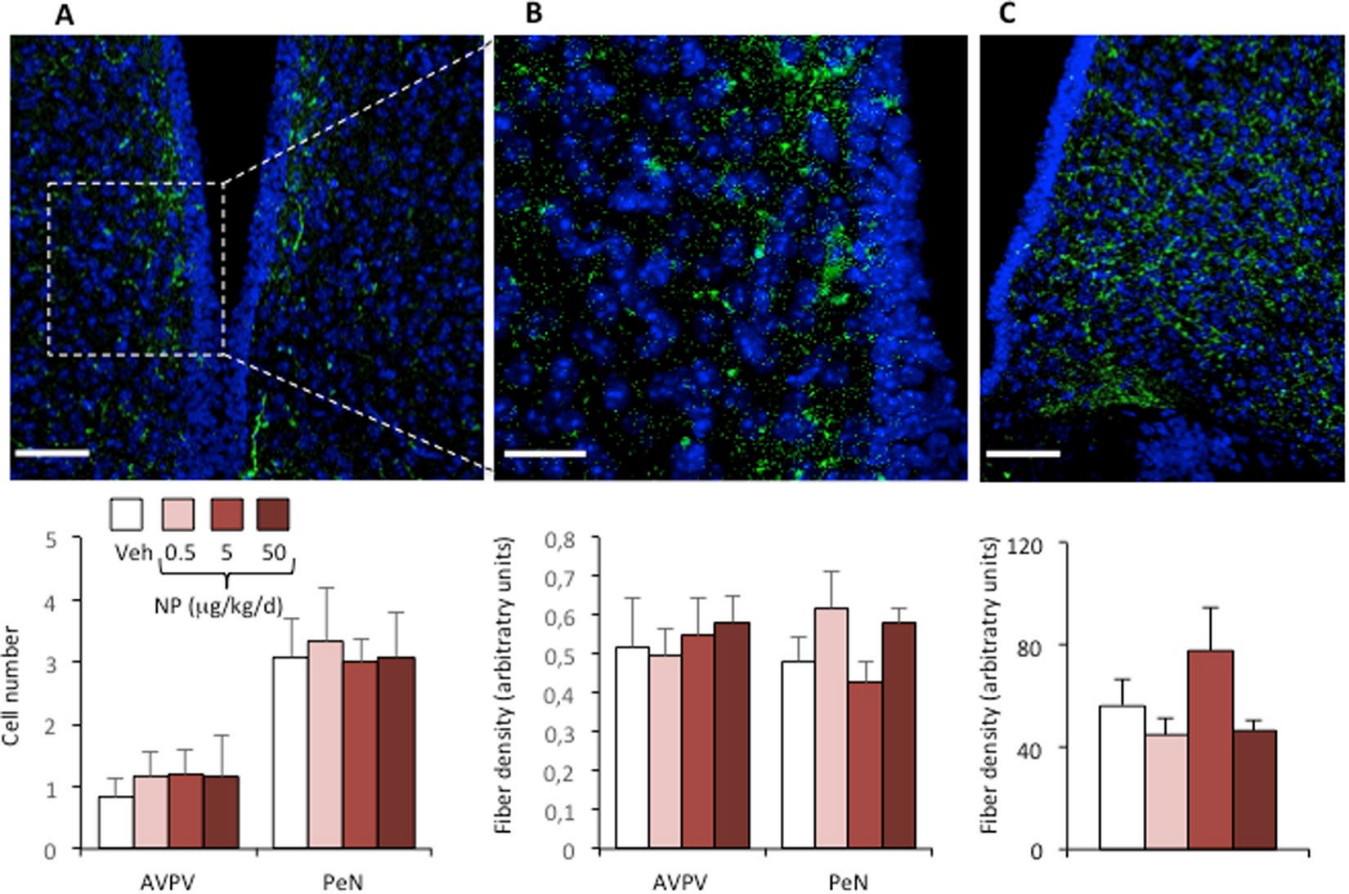
Kisspeptin immunoreactivity is unchanged following chronic exposure to NP. Mice were exposed to the vehicle or to NP at 0.5, 5, or 50 μg/kg/d. Analyses were performed in the rostroperiventricular area of the third ventricle and in the arcuate nucleus. (**A,B)** Representative immunolabeling (*upper panels*) in the periventricular nuclei (PeN) (**A**) with magnification of a kisspeptin-immunoreactive cell body (**B**) and quantification (*lower panels*) of the number of kisspeptin-immunoreactive neurons and fiber density per section in the anteroventral periventricular (AVPV) and PeN. (**C**) Fiber density in the arcuate nucleus. Data are expressed as the means ± S.E.M. of 5–6 males per treatment group. Scale bar = 50 μm (**A**,**C**) and 20 μm (**B**).

**Figure 2:**
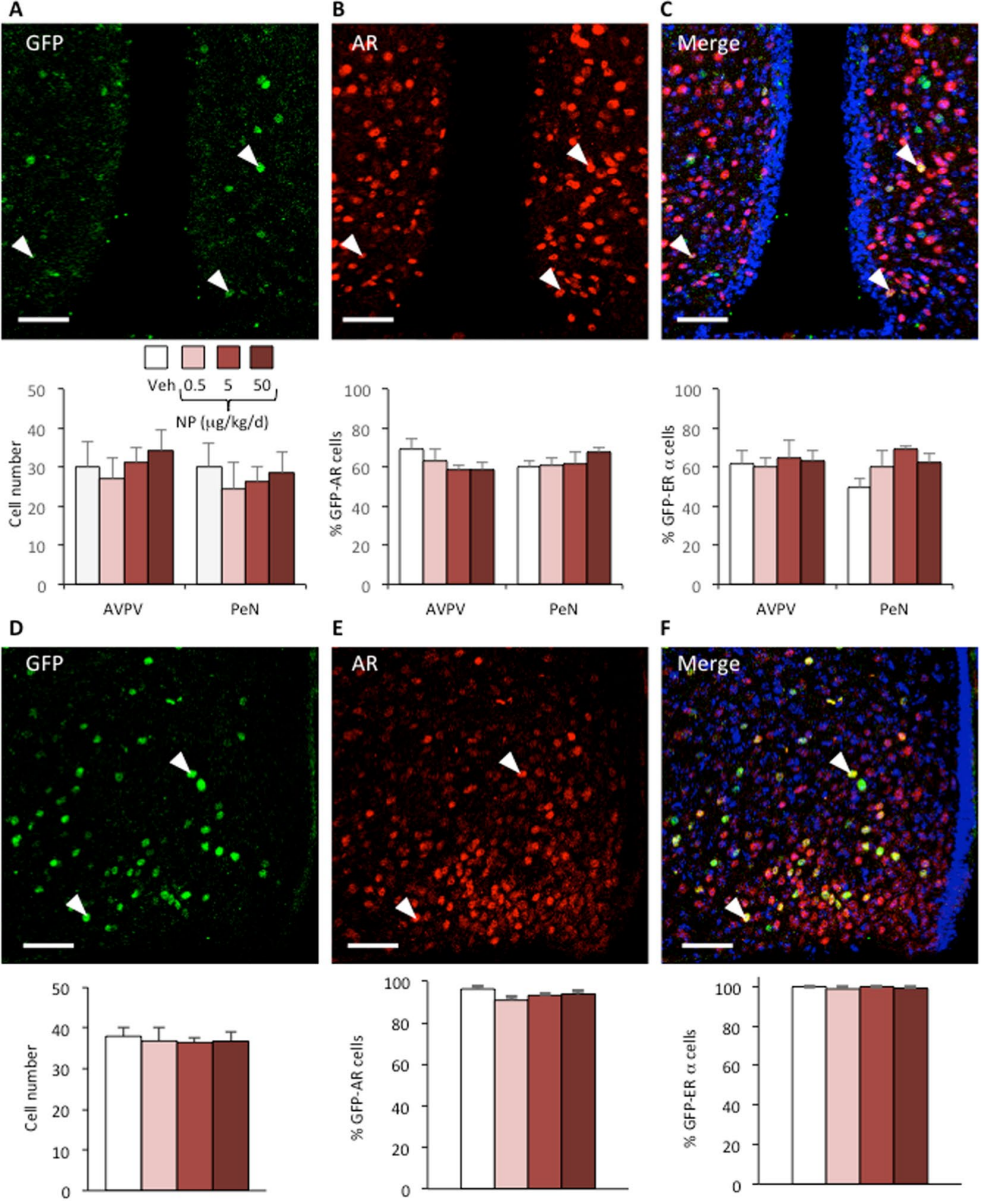
NP exposure does not affect GFP/AR or GFP/ERα immunoreactivity in mice expressing GFP under the control of the Kiss1 promoter. Mice were exposed to the vehicle (Veh) or NP (0.5, 5, or 50 µg/kg/d). Analyses were performed in the rostroperiventricular area of the third ventricle (RP3V) and arcuate nucleus. (**A**–**C**) Upper panels: Representative immunolabeling of GFP- (**A**) AR-immunoreactivity (**B**) and the merge (**C**) in the AVPV and PeN of Kiss1-creGFP males. White arrowheads show typical GFP and AR co-labelled cells. Scale bar = 50 μm. Lower panels: quantification of the number of GFP-cells (**A**) GFP/AR- (**B**) and GFP/ERα-co-expressing cells (**C**) per section in the AVPV and PeN. Data are expressed as the means ± S.E.M. of 3–4 males per treatment group. (**D**–**F**) Upper panels: Representative immunolabeling of GFP- (**D**) AR-immunoreactivity (**E**) and the merge shown by arrowheads (**F**) per section in the arcuate nucleus of Kiss1-creGFP males. White arrowheads show typical GFP and AR co-labelled cells. Scale bar = 50 μm. Lower panels: quantification of the number of GFP-cells (**D**), GFP/AR- (**E**), and GFP/ERα-co-expressing cells (**F**). Data are expressed as the means ± S.E.M. of 3–4 males per treatment group.

We investigated whether adult male mouse exposure to low doses of NP induces changes in the integrity of the HPG axis. We first measured the number of kisspeptin immunoreactive cells in the AVPV and PeN and the density of kisspeptin-immunoreactive fibers in the AVPV, PeN and arcuate nuclei. One-way analysis of variance (ANOVA) showed no significant effects of NP treatment on kisspeptin immunoreactivity in these regions of the brain (Fig. 1A–C). We further investigated possible alterations of *Kiss1* gene expression levels upon NP exposure by quantifying the number of GFP-positive cells in mice exposed to the vehicle or to NP from a line that expresses GFP under the control of the *Kiss1* promoter. One-way ANOVA showed no significant effects of NP treatment on the number of GFP-positive cells in the AVPV, PeN or the arcuate nuclei (Fig. 2A,D). The proportion of GFP-positive cells co-expressing the AR or ERα was also unchanged upon NP exposure (Fig. 2B,C,E,F). Kisspeptin and GFP immunoreactivity was also analyzed in the medial amygdala where a few Kiss1 cells have previously been detected in mice by *in situ* hybridization^26,27^ and kisspeptin immunoreactive cell bodies more recently in rat^28^. No significant immunolabelling was detected in either wild-type or Kiss1-CreGFP males (Figure S1), most likely due to very low expression levels.

The measurement of hormonal levels showed no effect of NP exposure on circulating testosterone levels (Table 1). Body weight and that of the androgen-dependent seminal vesicles and testes were comparable between the four treatment groups (Table 1).

**Table 1:** .

Urogenital tract weight and testosterone levels				
Exposure	Vehicle	NP-0.5	NP-5	NP-50
Body weight (g)	29.12 ± 0.49	29.30 ± 0.21	30.02 ± 0.48	29.06 ± 0.48
Testis weight (mg)	190.9 ± 7.64	204.9 ± 3.78	202.9 ± 4.15	193.4 ± 5.01
Testis weight (%bw)	0.66 ± 0.02	0.70 ± 0.01	0.68 ± 0.01	0.67 ± 0.02
SV weight (mg)	212.3 ± 11.15	224.2 ± 15.62	238.2 ± 13.85	234.3 ± 9.05
SV weight (%bw)	0.73 ± 0.04	0.77 ± 0.05	0.79 ± 0.05	0.81 ± 0.03
Testosterone (ng/ml)	1.40 ± 0.21	1.70 ± 0.34	1.40 ± 0.18	1.00 ± 0.19

Body weight (bw), testicular and seminal vesicles (SV) weights and testosterone levels in vehicle and males exposed to NP at 0.5, 5 and 50 μg/kg/d. Testicular and SV weights were also expressed as percentage of corresponding bw. Results are presented as means ± S.E.M. for 12 males per treatment group.

### Effects of chronic exposure to NP on male sexual behavior

#### Olfactory preference

**Figure 3:**
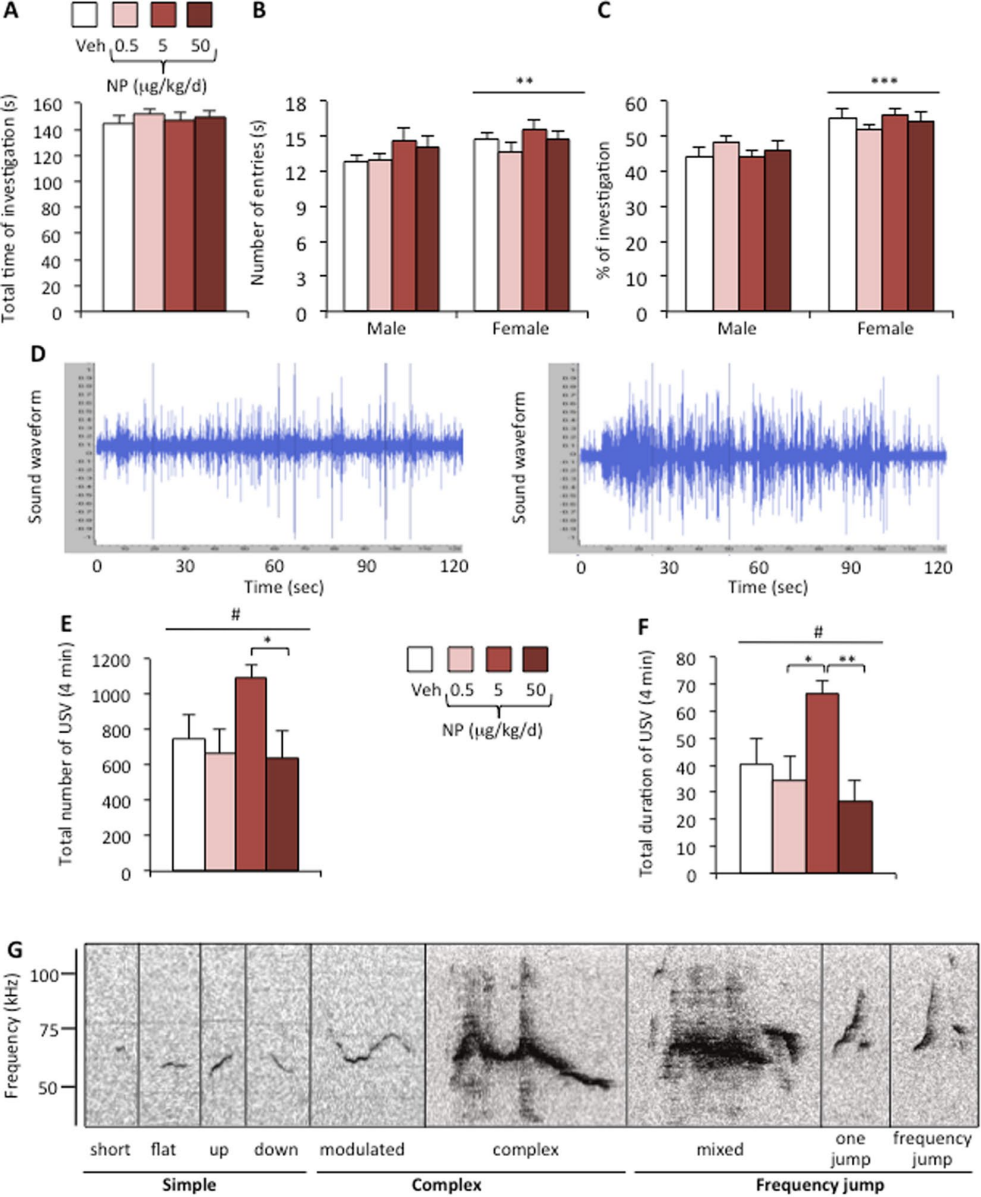
NP affects courtship vocalizations but not olfactory preference in male mice. (**A–C**) Olfactory preference was tested in a Y-maze. (**A**) Total time spent in the chemo-investigation of male and sexually receptive female stimuli by males exposed to the vehicle (Veh) or NP (0.5, 5, or 50 μg/kg/d). (**B**) Number of entries into the male or female arm of the Y maze. (**C**) Percentage of time spent in investigating males vs sexually receptive females. Data are expressed as the means ± S.E.M. of 12 males per treatment group, *p < 0.05 for the male arm. (**D**) Representative sound waveform for males exposed to vehicle (left) or NP at 5 μg/kg/d (right) in the presence of a sexually receptive female. (**E,F**) Total number (**E**) and duration (**F**) of ultrasonic vocalizations produced during the 4 min test by males exposed to the vehicle (Veh) or NP at 0.5, 5, or 50 μg/kg/d. Data are expressed as the means ± S.E.M. of 12 males per treatment group; One-way ANOVA (#) showed an effect of NP exposure. Post hoc comparisons are indicated**:** *p < 0.05, **p < 0.01, ***p < 0.001. (**G**) Representative ultrasonic vocalizations for each of the nine syllable types classified into three main categories (simple, complex, and with frequency jumps). Simple vocalizations were identified as syllables with unidirectional frequency with a duration shorter (short) or longer than 5 ms (flat, upward, and downward). Complex syllables were characterized by frequency modulations in more than one direction (modulated) or with the inclusion of one or more additional frequency components (complex). Frequency jumps were identified as syllables showing one (one jump) or several frequency jumps (frequency jump) with (mixed) or without sound during the transition.

We tested the ability of males to discriminate between male and female odors in olfactory preference tests using gonadally intact males vs sexually receptive females. The total time spent sniffing the two stimuli was equivalent for males from all four NP groups (Fig. 3A). Two-way ANOVA showed an effect of the stimulus on the number of entries into each arm of the maze (F_(1, 42)_ = 8.46, p = 0.006) and on the percentage of time spent sniffing each stimulus (F_(1,42)_ = 14.87, p = 0.0004), but NP exposure had no effect on either of the parameters (F_(3,42)_ = 1.29, p = 0.29 and F_(3,42)_ = 0.65, p = 0.59; respectively) (Fig. 3B,C). Males displayed a preference for female cues regardless of their exposure state.

#### Courtship vocalizations

The male produces ultrasonic vocalizations in response to female odors^29–31^. Vocalizations were thus recorded in the presence of a sexually receptive female. Male mice mainly vocalized at a frequency of 40–110 kHz. Figure 3D illustrates typical sound waveforms of vocalizations produced by a vehicle and an NP-5 exposed male. One-way ANOVA showed a significant effect of NP exposure on the total number (p = 0.042; Fig. 3E) and duration (p = 0.009, Fig. 3F) of emitted vocalizations. Post hoc analyses revealed a significant increase of the number and duration of ultrasonic vocalizations in the NP-5 group in comparison to the NP-50 group, and an increased duration in the NP-5 group in comparison to the NP-0.5 group.

**Figure 4:**
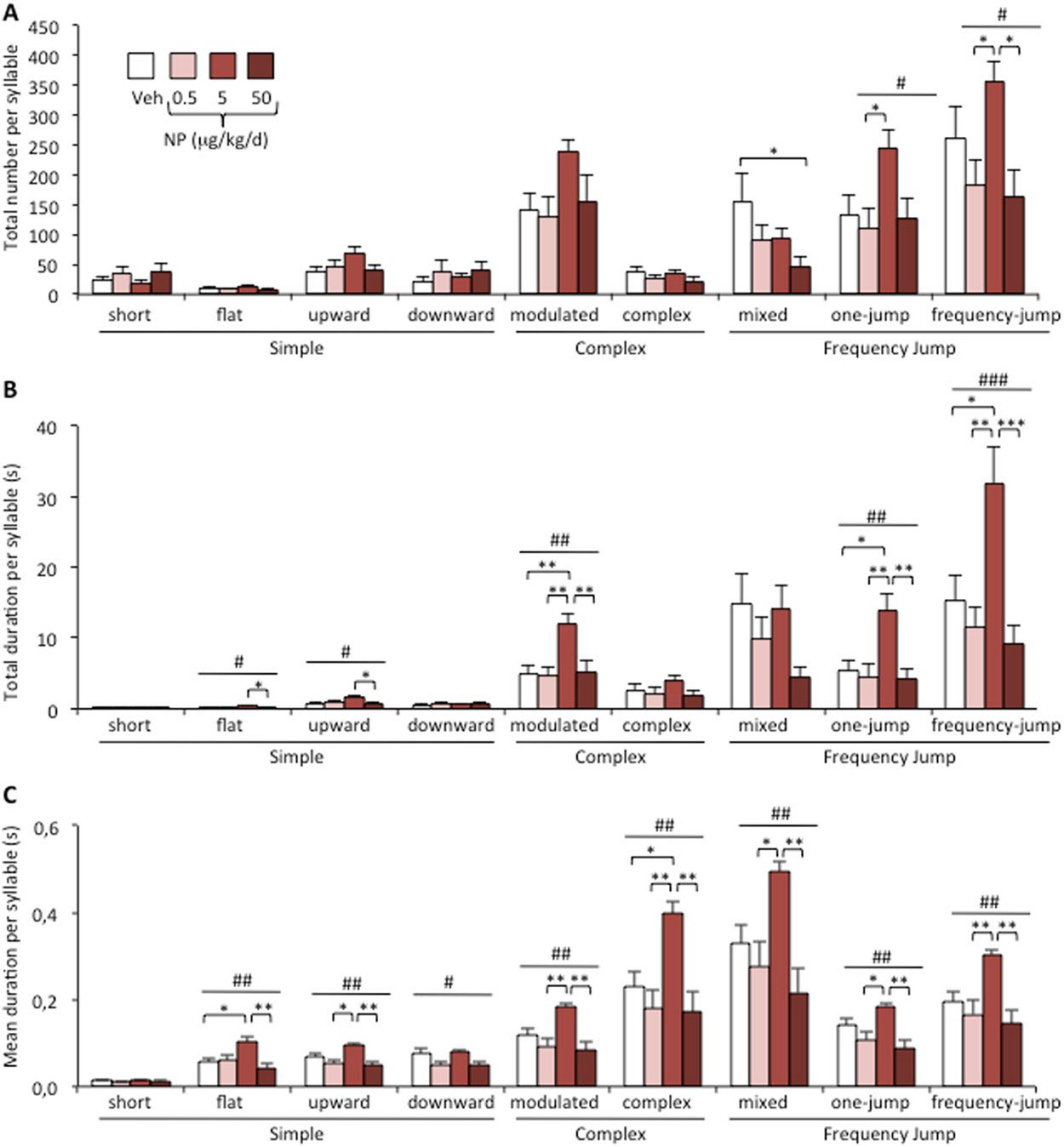
Qualitative analyses of syllables emitted by males exposed to NP. (**A**) Total number of each syllable type produced in the presence of a sexually receptive female during the 4 min recording for males exposed to the Veh or NP at 0.5, 5, or 50 μg/kg/d. One-way ANOVA (#) showed an effect of NP exposure on one jump (p = 0.037), and frequency jump syllables (p = 0.019). Post hoc comparisons are indicated**:** *p < 0.05, **p < 0.01, ***p < 0.001. Data are expressed as the means ± S.E.M. of 12 males per treatment group. (**B**) Total duration of each syllable type. One-way ANOVA (#) showed an effect of NP exposure on flat (p = 0.0398), upward (p = 0.028), modulated (p = 0.0019), one jump (p = 0.0015), and frequency jump syllables (p = 0.0003). Post hoc comparisons are indicated**:** *p < 0.05, **p < 0.01, ***p < 0.001. (**C**) Mean duration of each syllable type. One-way ANOVA (#) showed an effect of NP exposure on flat (p = 0.005), upward (p = 0.0049), downward (p = 0.027), modulated (p = 0.001), complex (p = 0.0019), mixed (p = 0.002), one jump (p = 0.002), and frequency jump syllables (p = 0.0017). Post hoc comparisons are indicated: *p < 0.05, **p < 0.01, ***p < 0.001.

Nine major syllables were identified (Fig. 3G) and grouped into three main categories identified as simple (short, upward, downward, flat), complex (modulated, complex), and with frequency jumps (mixed, one jump, with frequency jumps) as previously described^25,32^. A comparison of the total number of each syllable during the 4 min recording showed an effect of NP treatment on mixed, one-jump and frequency jump syllables of the frequency jump category (Fig. 4A). While a reduced number was observed for the mixed syllables in the NP-50 group, an increased syllable number was observed for NP-5 for one jump and frequency jump syllables in comparison to the other NP-exposed groups. NP exposure also affected the total duration of emitted syllables, with a significant effect on flat, upward, modulated, one jump, and frequency jump syllables (Fig. 4B). For all three categories, an increased total duration was observed for the NP-5 group in comparison to the vehicle or other NP doses. Figure 4C shows that the mean duration of flat, upward, modulated, complex, mixed, one jump and frequency jump syllables was increased for the NP-5 group. Therefore, chronic NP exposure affected the total number and duration of emitted ultrasonic vocalizations, with increased mean duration of eight of the nine emitted syllables.

#### Mating

**Figure 5:**
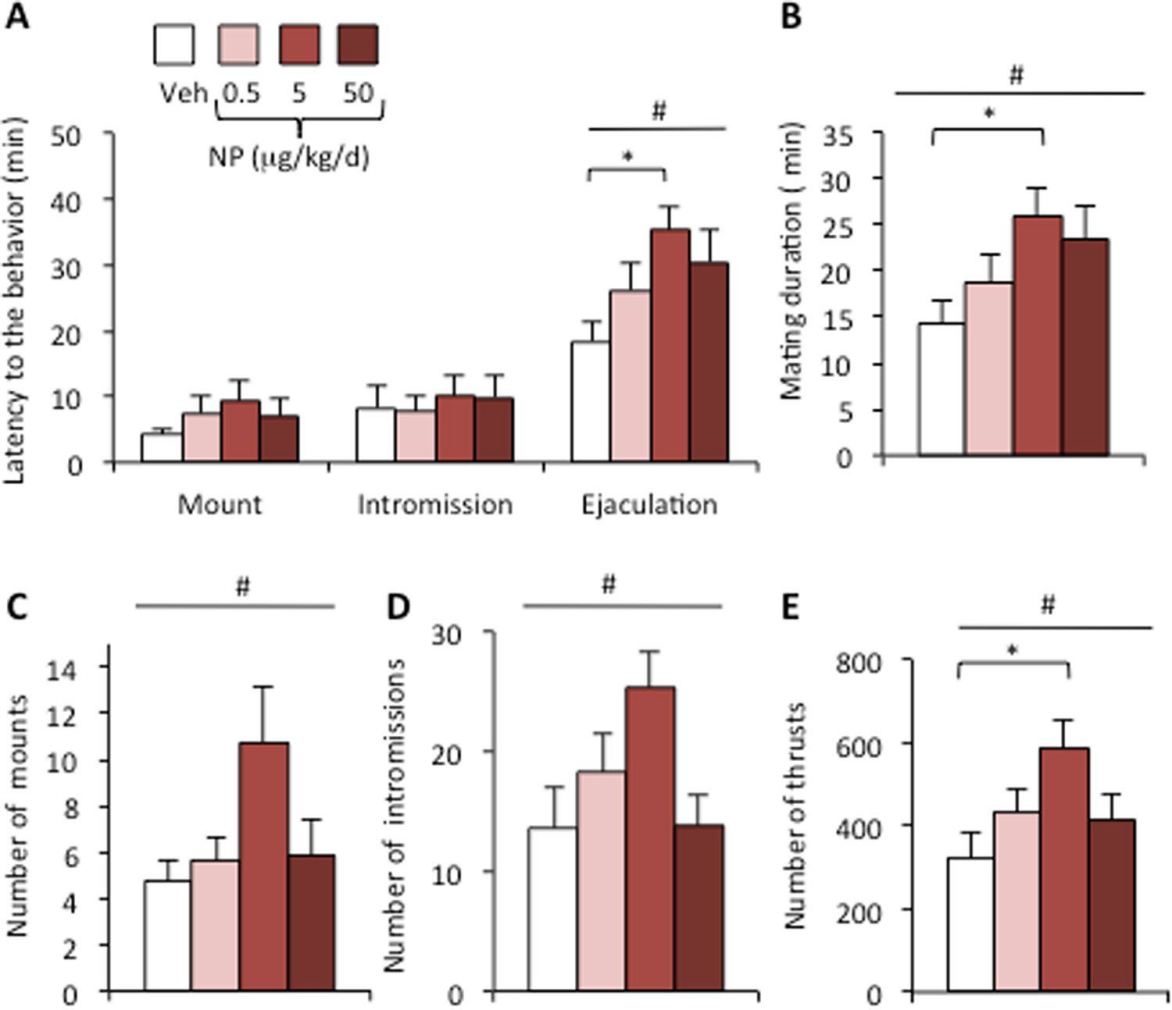
NP treatment delays the initiation of mating and increases mounts and thrusts frequency. (**A**) Latency to the first mount and intromission and to ejaculation in the mating test. (**B**) The time between the first mount and ejaculation (mating length) for males exposed to the vehicle (Veh) or NP at 0.5, 5, or 50 μg/kg/d. (**C–E**). Number of mounts (**C**), intromissions (**D**) and thrusts (**E**) exhibited by males. Data are expressed as the means ± S.E.M. of 12 males per treatment. One-way ANOVA (#) showed an effect of NP exposure; *p < 0.05 vs the vehicle group.

We compared the latency to the first mount, and intromission or ejaculation, between vehicle and NP-exposed male mice. One-way ANOVA showed no significant effect of NP exposure on the latency to the first mount (p = 0.62) or intromission (p = 0.59) as is illustrated in Fig. 5A. In contrast, a significant effect was observed on the latency to ejaculation (p = 0.03). Mice in the NP-5 group reached ejaculation later than those in the vehicle group (p < 0.05). The mating duration, calculated as the time from the first mount to ejaculation, was also increased in the NP-5 group in comparison to males exposed to the vehicle (Fig. 5B). There was also an effect of NP exposure on the number of mounts with and without intromission, and on the total number of thrusts (p < 0.05; Fig. 5C–E). Mice in the NP-5 group elicited these behaviors more frequently than those in the other groups did.

### Locomotor activity and anxiety-related behavior

**Figure 6:**
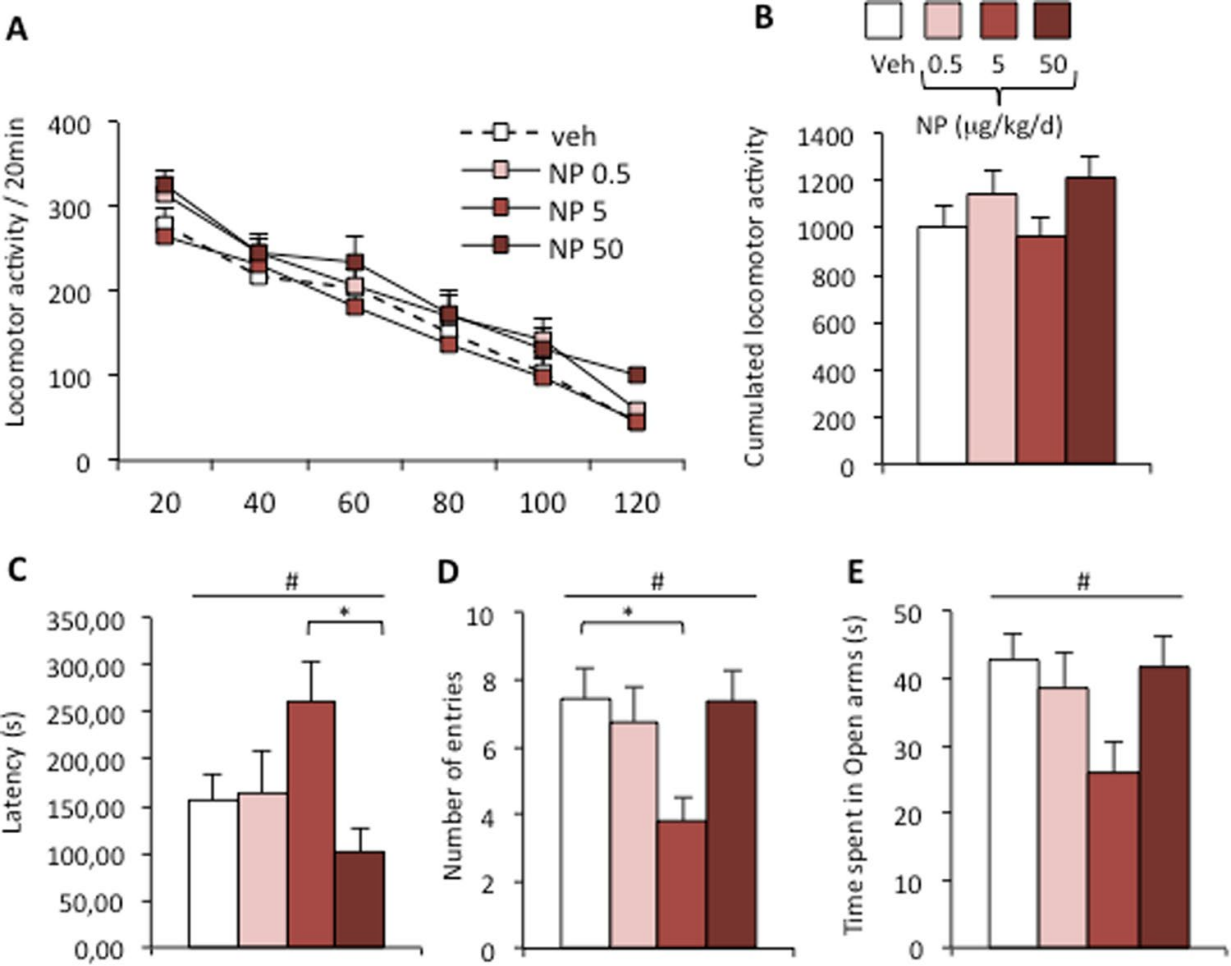
Anxiety, but not locomotor activity, is affected by NP exposure. (**A**) Spontaneous activity measured for 2 h by males exposed to the vehicle (Veh) or to NP at 0.5, 5, or 50 μg/kg/d. Data are expressed as the means ± S.E.M. of 12 males per treatment group. (**B**) Cumulative activity recorder during the 2 h test for the four treatment groups. (**C–E**) Anxiety-state level measured in the zero maze paradigm. Latency to the first entry in the open arm (**C**) the number of entries in the open arm (**D**) and time spent in the open arms (**E**) were analyzed. Data are expressed as the means ± S.E.M. of 12 males per treatment group. One-way ANOVA (#) showed an effect of NP exposure. Post hoc comparisons are indicated**:** *p < 0.05.

We next analyzed the effects of NP exposure on locomotor activity and anxiety-state level, since the alteration of these behaviors upon NP exposure could interfere with the expression of male sexual behavior. Two-way ANOVA of activity recorded for 2 h shows an effect of time (F_(5,220)_ = 118.63, p < 0.0001) but not of exposure (F_(3,220)_ = 1.53, p = 0.22; Fig. 6A). Similarly, no effect of treatment on cumulative locomotor activity was found (p = 0.22; Fig. 6B). In contrast, one-way ANOVA of the anxiety-state level measured in the zero maze paradigm showed an effect of NP exposure on the latency to the first entry and number of entries in the open arms (p = 0.024 and p = 0.034, respectively) as is shown in Fig. 6C,D. There was also an effect of DEHP exposure on the time spent in the open arms (p = 0.045; Fig. 6E). Post hoc analyses showed that males of the NP-5 group exhibited an increased latency before entering the open arms and a lower number of entries in comparison to males of the vehicle or other NP-exposed groups.

### Quantification of the number of AR- and ERα–immunoreactive cells in the neural circuitry that underlies sexual behavior

**Figure 7:**
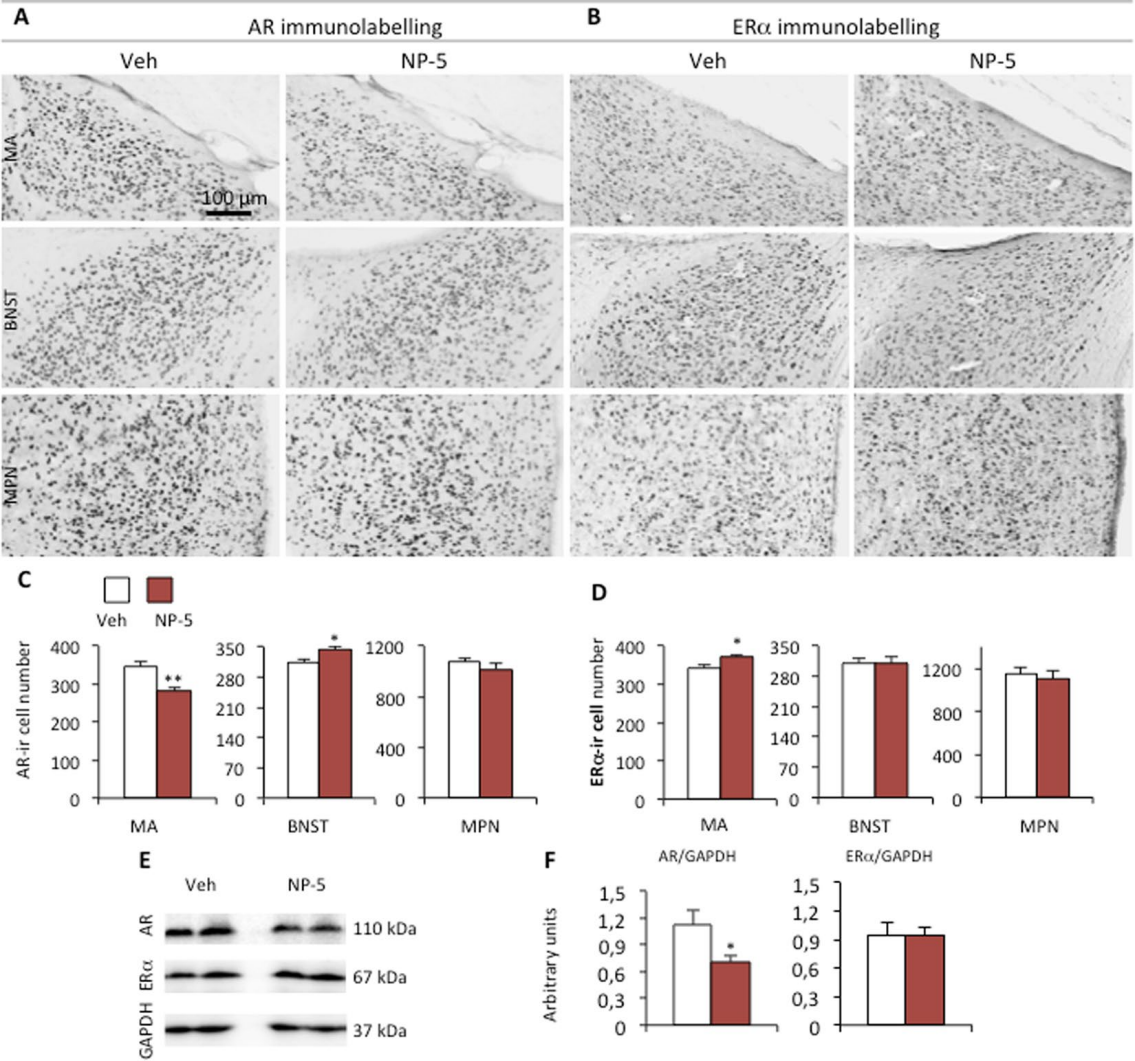
NP exposure affects the number of AR-ir and ERα-ir neurons and AR protein levels. (**A,B**) Representative AR- (**A**) and ERα-immunolabeling (**B**) in the posterodorsal medial amygdala (MA), bed nucleus of stria terminalis (BNST) and medial preoptic nucleus (MPN) of males exposed to the vehicle (Veh) or NP at 5 μg/kg/d. (**C,D**) Quantitative analyses of the number of AR- (**C**) and ERα-immunoreactive neurons (**D**) per section in the MA, BNST and MPN. Data are expressed as the means ± S.E.M. of 6 males per treatment group, *p < 0.05 compared to the vehicle group. (**E**) Representative Western blots of AR, ERα and GAPDH proteins in the hypothalamus of males exposed to the Veh or NP (5 μg/kg/d). (**F**) Quantification of the protein levels normalized to GAPDH. Data are expressed as the means ± S.E.M. of 7–8 males per treatment group, *p < 0.05 compared to the vehicle group.

NP exposure did not change testosterone levels but altered testosterone-dependent behavioral patterns. We therefore asked whether NP, in addition to its potential estrogenic activity, affected hormonal sensitivity of the neural structures involved in sexual behavior through modifications of sex steroid receptors. This analysis was conducted in chemosensory regions (medial amygdala, bed nucleus of stria terminalis and preoptic nucleus) where testosterone acts directly through AR activation or indirectly after neural metabolization into estradiol, which stimulates ERα^33–37^. We analyzed the effects of NP-5, since this dose triggers behavioral alterations on the number of neurons expressing these receptors. There were significantly fewer AR-immunoreactive cells in the medial amygdala of NP-exposed mice (−19%) than in vehicle-treated animals (Fig. 7A,C). In contrast, the number of AR-immunoreactive neurons in the bed nucleus of stria terminalis and ERα-ir neurons in the medial amygdala were increased (+10%) in comparison to the vehicle group (Fig. 7A–D). No changes were observed in the medial preoptic nucleus for the number of AR-ir or ERα-ir neurons (Fig. 7A–D). The medial preoptic nucleus represents the final brain area integrating chemosensory signals that are processed into behavioral responses. It plays a key role in the motivation to both vocalize and copulate^38–40^. We therefore performed a quantitative immunoblotting analysis in the hypothalamic region, for both AR and ERα proteins normalized to GAPDH (Figs 7E,F, S2). The data obtained show a significant reduction in the amount of AR protein (−26% compared to the vehicle group), but no changes for ERα.

## Discussion

The present study shows for the first time that chronic exposure of adult male mice to low doses of NP alters the expression of sexual behavior and increases anxiety-state level, without affecting the integrity of the HPG axis or testosterone levels. These changes, probably due to the estrogenic activity of NP, were associated with modifications in amounts of sex steroid receptor proteins in the neural circuitry that underlies sexual behavior. It is of great interest that almost all these effects were only observed at the NP dose of 5 μg/kg/d.

Exposure of adult male mice to NP affected the emission of ultrasonic vocalizations. In particular, the total number and duration of syllables were increased by exposure to NP at 5 μg/kg/d. Detailed analysis of the nine syllables emitted showed that these modifications were due to changes in the number of frequency jump syllables and to an increased mean duration of the majority of syllables. In order to assess the emission of ultrasonic vocalizations in mating conditions, recordings were performed in the presence of a sexually receptive female. Previous studies showed that, under such conditions, males emit the majority of produced ultrasonic vocalization while females vocalize in social interaction with females^41–45^. In particular, devocalization of males reduced ultrasonic vocalizations production to the level shown by devocalized male-female pairs^46^. In a more recent study using a new microphone array system to localize vocalizations from interacting mice, about 82% of vocal signal counts were attributed to males^47^. It is therefore reasonable to conclude that the observed vocalizing alterations derive mainly from changes in the behavior of NP-exposed males, although behavioral changes at the female level cannot be completely excluded.

Ultrasonic vocalizations are suggested to attract the receptive female and keep it near the male to facilitate copulation^8,48,49^. The analysis of copulatory behavioral patterns showed that increased emission of courtship vocalization in the NP-5 group was associated with normal latency to initiate the first mount and intromission, thereby suggesting that the precopulatory phase of sexual behavior was not affected. It must be probably difficult to appreciate the effects of increased vocalizations in our experimental conditions where the two partners are already very close in the cage. Once they started mounting and intromitting, males from the NP-5 group males attempted more mounts and thrusts and reached ejaculation later than males exposed to the vehicle. This lower sexual performance of NP-exposed males was associated with unchanged olfactory preference towards receptive females and general locomotor activity. In contrast, mice exposed to NP at 5 μg/kg/d exhibited an increased anxiety-state level measured by the zero maze test. Interestingly, chronic exposure of early pubertal male rats to NP (25 mg/kg) increased anxiety^50^, while acute exposure to lower doses (0.5, 5 mg/kg) had no effect^51^. The anxiety-related behavior of NP-exposed mice does not seem to be the main cause of sexual alteration since one would expect less interaction with the receptive female, a prolonged precopulatory phase and a lower rate of mounting and thrusting behaviors. It is possible that exposure to NP affects sexual behavior and anxiety-related behavior independently.

The observed behavioral alterations induced upon NP exposure were not due to changes in the HPG axis. Indeed, chronic exposure of adult male mice to NP did not alter kisspeptin immunoreactivity in the RP3V and arcuate nuclei. Co-expression of AR and ERα in kisspeptin neurons was also unaffected in NP-exposed male mice. In agreement with these data, levels of circulating testosterone and the weight of androgeno-sensitive seminal vesicles were unchanged by such a treatment. Previous studies showed that adult rat exposure to NP lowers testosterone levels^15,17,18^ and increases LH levels^15^. It is, however, important to note that this testosterone decrease was observed at NP doses ranging from 40 to 300 mg/kg/d, i.e. considerably higher than the NP doses used in the present study. In an analysis using the lower dose of 100 μg/kg, NP had no effect on basal levels of testosterone, although it lowered the increased levels induced by human chorionic gonadotropin^16^. It thus appears that low doses of NP do not affect the adult HPG axis.

The behavioral alterations described in the present work remind recent data on adult male rats showing that administration of estradiol delays ejaculation and increases the number of mounts^52^. Also, administration of estrogens to adult male mice is known to increase the emission of ultrasonic vocalizations in mice^53–56^. This suggests that NP mainly acts as an estrogenic compound, which is in good correlation with previous studies reporting such activity for this molecule in both *in vitro* and *in vivo* systems^57^. It is interesting to note that this potential estrogenic effect of NP was associated with changes in the expression level of sex steroid receptors. In the medial amygdala of males exposed to NP at 5 µg/kg/d, the number of ERα-immunoreactive cells was increased while that of AR-immunoreactive cells was reduced. Such opposite changes in the expression level of these two receptors were previously reported in other models. For instance, deletion of the neural *AR* in male mice resulted in an increased number of ERα-immunoreactive cells and was associated with sexual deficiency^24,36^. Increased ERα-immunoreactivity was also observed in the medial amygdala of bisphenol A-exposed mice, which exhibited sexual deficiency^24^. In the preoptic nucleus of NP-exposed males mice, quantification by Western blotting of AR showed a similar down-regulation without changes in ERα levels, while an increase in AR-immunoreactive cells was observed in the bed nucleus of stria terminalis. Whether NP elicits directly or indirectly these area-specific modifications in AR and ERα levels remain to be studied. It is, however, important to emphasize that these changes following exposure to a low dose of the estrogenic NP compound can participate in further disrupting the activation of sexual behavior by testosterone and its neural metabolite estradiol.

The effects induced by adult mice exposure to NP were almost exclusively induced at the dose of 5 μg/kg/d. No significant effects were seen at 0.5 or 50 μg/kg/d, suggesting non-monotonic induced effects of NP. Several studies reported non-monotonic effects of endocrine disrupters exhibiting estrogenic activity, similar to those triggered by endogenous hormones, with more effects observed at low relevant doses^58^. For instance, we previously described a non-monotonic response on male behavior induced by adult exposure to bisphenol A, with the TDI dose inducing sexual deficiency compared to the no-observed adverse effect level dose^24^.

The NP dose that elicits significant changes in male behavior in the present paper corresponds to the TDI dose value derived by the Danish Institute of Safety and Toxicology^59^. The calculation of reference doses for NP should, therefore, be updated in light of recent studies. The NP dose of 5 μg/kg/d also corresponds to the average exposure to environmental sources estimated by the EU^60^. This means that environmental exposure to NP presents a risk to both wildlife and humans, given the well-conserved hormonal regulation of courtship vocalizations and mating in several vocalizing species, along with libido in humans.

In conclusion, chronic exposure of adult male mice to low doses of NP alters the emission of courtship vocalizations and increases the latency to ejaculation as well as the rate of mounting and thrusting. The anxiety state level of exposed mice was also increased. The dose of 5 μg/kg/d was sufficient to induce virtually all behavioral modifications observed; thus NP is acting through induction of a non-monotonic dose response. These behavioral alterations are associated with unchanged integrity of the HPG axis and circulating levels of testosterone. In addition to its potential estrogenic activity, NP triggered changes in the amount of the neural AR and ERα proteins, indicating NP-induced endocrine disruption of the neural circuitry involved in sexual behavior. These data, taken together with our previous findings for bisphenol A and phthalates highlight the vulnerability of the adult brain to exposure to endocrine disrupters.

## Methods

### Animals and Treatment

Analyses were performed according to European legal requirements (Decree 2010/63/UE) and were approved by the “Charles Darwin” Ethical committee (project number 01490-01). Mice were housed in nest-enriched polysulfone cages maintained at 22 °C, with a 12:12 h light-dark cycle, and were fed a standard diet with free access to food and water as previously described^24^. The numbers of experimental and control groups are given in the figure legends.

Eight-week-old C57BL/6J males (Janvier) were acclimatized for two weeks. Kiss1-creGFP knock-in mice (Kiss-1tm1.1(cre/EGFP)Stei/J; The Jackson Laboratory) were bred on a C57BL/6J background for more than 10 generations. To mimic the major route of exposure to NP, mice were fed ad libitum a standard diet containing the vehicle (control group) or contaminated by NP (a mixture of ring and chain isomers; Sigma-Aldrich #290858). This compound was dissolved in an ethanol-water mix and incorporated into the food, so that the exposure was equivalent to 5 (NP-5 group), a lower dose of 0.5 μg/kg/d (NP-0.5 group) or a higher dose of 50 μg/kg/d (NP-50 group). NP doses were calculated for a daily intake food of 5 g per animal. As the no observable adverse effect level (NOAEL) dose of NP differs in various studies, the chosen doses were based on estimated average exposure to environmental sources of NP (5 μg/kg body weight/d), with 70–80% of this contamination attributed to fish and shell consumption^60^. This dose also corresponds to the tolerable daily intake (TDI) value derived by the Danish Institute of Safety and Toxicology^59^.

Mice were weighed weekly and NP doses adjusted to their body weight. This latter parameter, followed throughout the exposure period, was similar in the four treatment groups (mean of 26.21 ± 0.35 g on the first day of exposure and 30.3 ± 0.51 g on the last day of exposure). Analyses were started after 4 weeks of exposure to NP, which was maintained during the whole experimental period.

### Immunohistochemistry

Animals (5–6 C57BL/6J and 3–4 Kiss1-CreGFP males per treatment group) were euthanized and transcardially perfused with a solution of 4% paraformaldhehyde (PFA) in phosphate buffer. Brains were post-fixed overnight in 4% PFA, cryoprotected in sucrose and stored until analyses. Brains were sliced into coronal sections of 30 µm and processed for single and double immunolabeling.

Kisspeptin immunolabeling was processed with anti-kisspeptin AC053^61^ and sections were counterstained with Hoechst as previously shown^62^. The number of labeled cell soma and fiber density in the AVPV and PeN of the rostral preoptic periventricular nucleus^4^ and arcuate nucleus were measured. Image for fiber were acquired with LSM 700 confocal microscope (Zeiss) under x40 magnification. Quantifications were performed on three sections sampled at one anteroposterior level of the RP3V corresponding to the AVPV (plate 29) and two level corresponding to the PeN (plates 30 and 31–32 of Paxinos and Franklin atlas) and on three sections of the arcuate nucleus sampled at the level of the anterior, median and caudal arcuate nucleus (plates 43, 47 and 50)^63^.

For EGFP/ERα and EGFP/AR double immunolabelling, sections were blocked for 30 min with 2% normal donkey serum, and incubated with polyclonal chicken anti-GFP (1:10000, Table 2) and either rabbit anti-AR or anti- estrogen receptor (ER)α (1:250 for both, Table 2) for 3 days. A cocktail of alexafluor 488-conjugated donkey anti-chicken and cyanin 3-conjugated donkey anti-rabbit antibodies (1:500 for both, Table 2) was applied to the sections for 2 h. Sections were counterstained with Hoechst and mounted on slides with fluoromount (Southern Biotechnologies). Images were acquired with LSM 700 confocal microscope (Zeiss) under x20 magnification and x0.7 digital zoom. Quantifications of the proportion of GFP cells coexpressing either AR or ERα (Table 2) were performed in one anteroposterior level of the RP3V corresponding to the AVPV (plate 29) and one level corresponding to the PeN (plate 31–32 of Paxinos and Franklin atlas) and from 12 equidistant (120 μm) hemi-sections encompassing the whole rostro-caudal extent of the arcuate nucleus (plates 41 and 53). Data are displayed as average numbers of GFP-positive nuclear counts per section or hemi-section and per group.

**Table 2:** .

Primary antibodies	Dilution	Catalog #	Provider
Sheep anti-kisspeptin	1:10000	AC053	Home^61^
Chicken anti-GFP	1:10000	GFP-1020	Aves Lab
Rabbit anti-AR	1:250 (IF), 1:200 (IHC)	AR (N-20) sc-816	Santa Cruz Biotechnology
Rabbit anti-(ER)α	1:250 (IF), 1:200 (IHC)	ERα (MC-20) sc-542	Santa Cruz Biotechnology
**Secondary antibodies**			
AF488 Donkey anti-sheep	1:1000	713-545-147	Jackson ImmunoResearch
AF488 Donkey anti-chicken	1:500	703-545-155	Jackson ImmunoResearch
Cy3 Donkey anti-rabbit	1:500	711-165-152	Jackson ImmunoResearch
Biotin SP Goat anti-rabbit	1:200 (AR), 1:500 (ERα)	111-065-003	Jackson ImmunoResearch

Antibodies used in immunohistochemistry (IHC), immunofluorescence (IF) and Western blotting analyses.

AR- and ERα-immunolabeling was processed as previously shown^37^. Images were acquired with Nikon Eclipse 80i light microscope under x10 magnification. The number of labeled cells per sections was counted in anatomically matched sections identified using the Mouse Brain Atlas of Paxinos and Franklin. Counting surface for medial amygdala (plate 45), bed nucleus of stria terminalis (plate 33) and medial preoptic nucleus (plate 30) were respectively 0.07 mm^2^, 0.055mm^2^ and 0.31mm^2^.

### Urogenital tract weight and hormone measurements

Animals (12 males per treatment group) were euthanized between 9:00 to10:00 AM to collect their blood and to weigh testes and seminal vesicles. Serum was collected and circulating levels of testosterone were measured by RIA at the hormonal assay platform of the laboratory of behavioral and reproductive physiology (UMR 7247 INRA/CNRS/Université François Rabelais) using ^3^H-T, as previously described^24^. The mean intra-assay coefficient of variation was 7% and the assay sensitivity was 125 pg/ml.

### Behavioral tests

Tests were conducted under red-light illumination 2 h after lights were turned off and were videotaped for later analysis. Four weeks after exposure to NP, naïve males (12 per treatment group) were housed individually for 3 days. Each male was then paired in its home cage with a sexually receptive female, prepared as described below, and the males were allowed to reach ejaculation. This first sexual experience was performed to increase behaviors such as olfactory preference, ultrasonic vocalizations and sexual interest of the males studied toward receptive females^24,64,65^. Behavioral tests were conducted two weeks later. Analyses of recordings were performed by blind observers since males were identified by numbers given at weaning, without any information on their exposure state.

#### Preparation of sexually receptive females

C57BL/6J females used as stimuli were ovariectomized under general anesthetic (xylazine/ketamine) and implanted with SILASTIC implants (Dow Corning, Midland, MI) filled with 50 µg of estradiol-benzoate (Sigma-Aldrich) in 30 µl of sesame oil. Four to five h before the tests, females were given a sub-cutaneous injection of 1 mg of progesterone (Sigma-Aldrich) in 100 µl of sesame oil. Female receptivity was verified before the beginning of experiments using sexually experienced males.

#### Olfactory preference test

Olfactory preference was assessed in an enclosed plexiglas Y-maze as previously described^24^. On the day of the test, male mice were offered the choice between an anesthetized sexually receptive female and an anesthetized gonadally intact male. The time spent in chemo-investigation of each stimulus and the number of entries into each arm of the Y-maze were scored during a 5 min test.

#### Ultrasonic vocalization recording

Each male was tested in its home cage, in the presence of a sexually receptive female. After the introduction of the female, vocalizations were recorded for 4 min with a microphone (UltraSoundGate), which was connected to an ultrasound recording interface plugged into a computer equipped with the recording software Avisoft-SASLab Pro 5.2.09, then analyzed using SASLab Pro (Avisoft Bioacoustic). Spectrograms were generated for each call detected (frequency resolution: FFT-length: 512; frame size: 100%; overlap: 50%). The parameters used for the automatic quantification of the ultrasonic vocalizations were: cut-off frequency of 30 kHz, element separation based on an automatic single threshold with a hold time of 15 ms. The nine major syllables were grouped into three main categories identified as simple (short, upward, downward, flat), complex (modulated, complex), and with frequency jumps (mixed, one jump, with frequency jumps) as previously described^25,32^. The total number and duration of vocalizations and the number and duration of each syllable were analyzed.

#### Mating

Each male was tested in its home cage for 10 h after the introduction of the receptive female as previously described^24^. The latency to the first intromission or ejaculation (time from female introduction into the cage until the behavioral event), the mating duration (time from the first intromission until ejaculation), the frequency of mounts with and without intromissions, as well as the intromission ratio were scored.

#### Locomotor activity

Activity was analyzed in a computed circular corridor as previously described^36^. Briefly, the male subject was introduced into a circular corridor made up of two concentric cylinders crossed by four diametrically opposite infrared beams (Imetronic). The locomotor activity was counted when animals interrupted two successive beams and had thus traveled a quarter of the circular corridor. Spontaneous activity was recorded for 2 h and was expressed as cumulative activity every 20 min.

#### Anxiety-related behavior

The elevated zero maze test was conducted as previously described^66^. Males were placed in the closed arms and were allowed to explore the maze freely for nine min. The latency time before entry into the open arms and the time spent in the open arms were analyzed. A mouse was considered to be in an open arm once all its 4 paws had entered. The light intensity was 60 lux.

### Western blotting

Protein extracts (30 μg), prepared from 7–8 males per treatment group as previously described^66^, were separated on a 10% polyacrylamide gel, and transferred onto a nitrocellulose membrane. As the specificity of antibodies used was verified in previous studies^36,55,59^, the membrane was cut in order to perform the three quantifications on the same loaded samples (Figure S1). Blots were probed overnight with polyclonal anti-AR (1:400, Santa-Cruz Biotechnology), anti-ERα (1:400, Santa-Cruz Biotechnology) or anti-glyceraldehyde 3-phosphate dehydrogenase gene GAPDH (1:10000, Santa-Cruz Biotechnology) and then with peroxidase-conjugated second antibodies diluted at 1:5000 (Amersham Bioscience).

Signals were visualized with the Super Signal detection kit (Thermo Scientific), quantified with ImageJ software (NIH) and normalized with respect to GAPDH.

### Statistics

Data were expressed as the mean ± S.E.M. Student’s t-tests were used to determine the effects of NP exposure on kisspeptin immunoreactivity, and on the number of AR- and ERα-expressing cells. Two-way ANOVA was used to analyze the main effects of NP exposure and stimulus on olfactory preference, and time on locomotor activity. One-way ANOVA was used to analyze the effect of NP exposure on the remaining data. Tukey post-hoc tests were used to determine group differences. P values of less than 0.05 were considered to be significant.

### Data Availability

The datasets generated during and/or analyzed during the current study are available from the corresponding author on reasonable request.

## Electronic supplementary material

Supplementary material
