# Supplementary material for: Adult male mice exposure to nonylphenol alters courtship vocalizations and mating
Source: Sci Rep. 2018 Feb 14;8:2988. doi: 10.1038/s41598-018-21245-9 (PMC5813014; doi:10.1038/s41598-018-21245-9)
Supplement: Supplementary file 1 — Supplementary material [file 41598_2018_21245_MOESM1_ESM.doc]

**Supplemental Material**

Adult male mice exposure to nonylphenol alters courtship vocalizations and mating

Daphné Capela, Carlos Dombret, Kevin Poissenot, Manon Poignant, Aude Malbert-Colas, Isabelle Franceschini, Matthieu Keller, Sakina Mhaouty-Kodja

**Table of contents**

Figure S1

Figure S2

**Figure S1**. **A-B**. No kisspeptin nor GFP immunoreactivity was detected in the medial amygdala of respective wild-type (**A**) and Kiss1-creGFP males (**B**). Scale bar = 100 μm.

**Figure S2**. Western blots of AR, ERα and GAPDH in the hypothalamus of the Veh and NP-5 groups. The same membrane was cut in order to perform immunolabeling for AR, ERα and GAPDH on the same loaded samples as described in Material & Methods.
